# The interplay between epitranscriptomic RNA modifications and neurodegenerative disorders: Mechanistic insights and potential therapeutic strategies

**DOI:** 10.1002/ibra.12183

**Published:** 2024-11-11

**Authors:** Muhammad Abu Talha Safdar Hashmi, Hooriya Fatima, Sadia Ahmad, Amna Rehman, Fiza Safdar

**Affiliations:** ^1^ Department of Zoology University of Central Punjab Lahore Pakistan; ^2^ Institute of Zoology University of Punjab Lahore Pakistan; ^3^ Department of Biochemistry University of Narowal Narowal Pakistan

**Keywords:** Alzheimer's disease, epitranscriptomics, neurodegeneration, Parkinson's disease, RNA modifications

## Abstract

Neurodegenerative disorders encompass a group of age‐related conditions characterized by the gradual decline in both the structure and functionality of the central nervous system (CNS). RNA modifications, arising from the epitranscriptome or RNA‐modifying protein mutations, have recently been observed to contribute significantly to neurodegenerative disorders. Specific modifications like N6‐methyladenine (m6A), N1‐methyladenine (m1A), 5‐methylcytosine (m5C), pseudouridine and adenosine‐to‐inosine (A‐to‐I) play key roles, with their regulators serving as crucial therapeutic targets. These epitranscriptomic changes intricately control gene expression, influencing cellular functions and contributing to disease pathology. Dysregulation of RNA metabolism, affecting mRNA processing and noncoding RNA biogenesis, is a central factor in these diseases. This review underscores the complex relationship between RNA modifications and neurodegenerative disorders, emphasizing the influence of RNA modification and the epitranscriptome, exploring the function of RNA modification enzymes in neurodegenerative processes, investigating the functional consequences of RNA modifications within neurodegenerative pathways, and evaluating the potential therapeutic advancements derived from assessing the epitranscriptome.

## Graphical abstract

This review elucidates the pivotal role of RNA modifications in the etiology and progression of neurodegenerative disorders. RNA modification proteins (RMPs) including FTO, HNRNPA2B1, ADAR1/2, METTL3/14, NSUN2, and ALKBH5 are implicated in initiating dysregulated RNA modifications, thereby disrupting RNA metabolism. Perturbed RNA metabolism leads to compromised RNA stability, mRNA splicing irregularities, and impaired RNA transport. Neurodegeneration ensues as a consequence of these disruptions, manifesting in altered cellular functions within the brain. These include impaired translation efficiency, dysregulated gene expression, compromised neurotransmitter synthesis and release, aberrant cell proliferation, dysfunctional stress response, disrupted energy metabolism, impaired cellular communication, neuroinflammation, and protein aggregation. Promising therapeutic avenues include antisense oligonucleotides and small molecule inhibitors, which offer potential mitigation of neurodegenerative effects by targeting RNA modifications. These therapeutic strategies hold the promise of restoring cellular functionality and ameliorating the progression of neurodegenerative diseases, offering hope in combating these debilitating conditions.

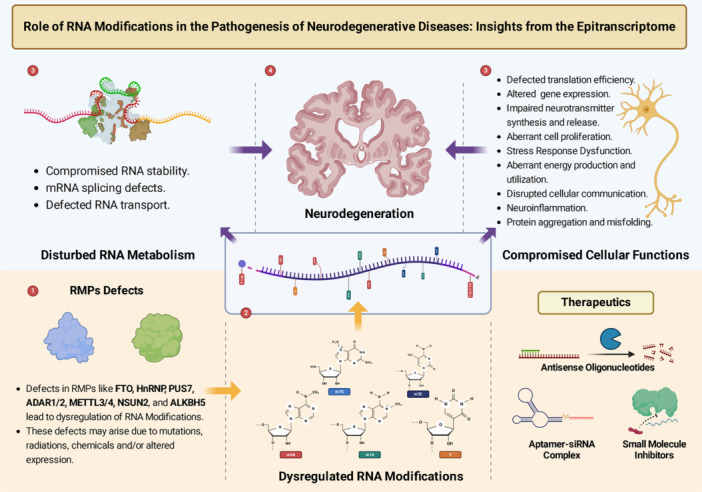

## 1 INTRODUCTION

Neurodegenerative disorders are an age‐related group of disorders categorized by the progressive deterioration of the structure and function of the central nervous system (CNS). The most important pathological types of neurodegenerative disorders are the breakdown of the blood‐brain barrier (BBB) and the aggregation of proteins.^1^ Some of the most common neurodegenerative disorders are Alzheimer's disease (AD), amyotrophic lateral sclerosis (ALS), and Parkinson's disease (PD). Neurodegenerative disorders rank as the 7th highest cause of mortality worldwide.^2^ In the coming years, there is an anticipated increase in the number of neurodegenerative patients due to the aging population. Unfortunately, there is no effective cure or potential therapies to control these overwhelming disorders.^1^ AD is the most prevalent neurodegenerative disease, impacting 10–30% of people aged 65 and older, with an annual incidence rate of 1–3%.^3^ AD is caused by the degeneration of neuronal cells, which is also the main cause of dementia that affects the thinking and independence of personnel's daily routine. Further risk factors such as head injuries, vascular disorders, increasing age factors, genetic factors, and environmental aspects also play a role in disease development.^4^ PD is the second most prevalent and progressively advancing neurodegenerative condition disorder, characterized by prominent symptoms such as bradykinesia, compromised posture, and tremors.^5^ ALS is a worldwide disorder that has a detrimental effect on the quality of life of patients and places a significant problem on both families and society at a large scale.^6^ ALS mainly affects both upper and lower motor neurons which leads to gradual paralysis and ultimately death from respiratory failure.^7^ Neurodegenerative disorders are serious problems all over the world. There are many mechanisms involved in the progression of various neurodegenerative disorders.

RNA plays a significant role in transcription and translation, the process involved in the expression of genes.^8^ There are three classes of RNA, in which messenger RNA (mRNA) links with transcription, transfer RNA (tRNA) assists in decoding the mRNA, and ribosomal RNA (rRNA) is associated with ribosomes to make proteins.^9^ Current studies have shown how abnormal functioning of RNA such as dysfunction of RNA transport, splicing stabilization microRNAs (miRNA) or tRNA biogenesis contributed to age‐related neurodegenerative disorders.^10^, ^11^, ^12^ Altered RNA metabolism participates in neurodevelopmental disorders and neurodegenerative disorders, with binding proteins playing a significant role in both types of pathogenesis.^11^, ^13^ As documented by the MODOMICS database, 170 RNA alterations have been identified, and some of these major modifications have been observed to be directly linked with neurological disorders.^14^ Changes and mutations in the RNA‐modifying proteins have also been associated with several disorders, including obesity, infertility neurodegenerative and neurodevelopmental disorders, and cancer.^15^, ^16^ The splicing mechanism of mRNA plays a significant role in neuronal transcription complexity, structure‐function, shaping, and differentiation processes.^17^, ^18^, ^19^

Recent studies have demonstrated that RNA modifications like alternative polyadenylation (APA), N6‐methyladenosine modification (m6A), N5‐methylcytosine modification (m5C), N1‐methyladenosine modification (m1A), pseudouridine modification, and adenosine‐to‐inosine (A‐to‐I) editing contribute to the pathogenesis of neurodegenerative disorders. APA of mRNA is used mainly for multiple polyadenylation process transcripts and conjugation along with alternative splicing. APA develops the mRNA isoforms to increase cellular diversity.^20^ APA is a universal process in eukaryotes and mammals and approximately 70% of mRNA‐encoding genes come under the control of APA.^21^, ^22^, ^23^ To maintain RNA equilibrium, mRNA is transcribed with the nucleus through different biological mechanisms influenced by the cis‐acting elements. Exonucleases and endonucleases participate in the deterioration of the process.^24^ The addition of methylation capping at the 5' of theuntranslated region (UTR) and polyadenylation at 3' UTR protects the mRNA from these nucleases. The expression of genes depends upon the stability of mRNA, which is calculated by the half‐life of mRNAs.^25^ The half‐life of RNAs can be influenced by a variety of processes leading to its reduction or extension.^26^ Recent research has investigated ribosome assembles at the distal end of the axon, but previous understanding shows that ribosomal protein is translated at the proximal end and then transported within messenger Ribonucleoprotein (mRMP) complexes to the distal site.^27^, ^28^ One of the 170 + RNA modifications discovered so far is, m1A. m1A was first time identified on tRNA at the position of 9, 14, and 58, which is also important for 3D structure and stability due to its positive charge, aiding in proper folding.^29^, ^30^, ^31^ Recent findings show that reversible regulation of m1A by tRNA m1A methyltransferase (MTase), is accomplished by AlkB Homolog 1 (ALKBH1) and AlkB Homolog 3 (ALKBH3). ALKBH1‐mediated demethylation decreases translation initiation and usage of demethylated tRNAs in protein synthesis.^30^, ^32^ Another RNA modification, 7‐methylguanosine (m7G) is present in 46 positions on a variable loop of tRNA mounted by tRNA m7G46 methyltransferase.^33^, ^34^ This change produces a tertiary base pair with C13 and G22, that contributes to stabilizing the tRNA structure.^35^ Pseudouridine is the most common type of RNA‐modified nucleoside in all kinds of RNA.^36^ This modification induces changes in the secondary structure by increasing base stacking, generating more rigid sugar‐phosphate backbones, and refining base pairs.^37^ Within tRNA, m5C is located at the positions of 34, 38, 40, 48, 49, and 50.^38^ Notably, m5C at positions 48 and position 49 are situated in the T‐loop of tRNA and participate in the stability of tRNA as well as protein production.^38^

Dysregulation of RNA modification leads to a wide spectrum of disorders spanning from cancer to neurodegenerative disorders.^39^ In this review, we find several thrilling studies, reporting the impact of RNA modification like m5C, m6A, m1A, A‐to‐I editing, and pseudouridine, along with the broader field of epitranscriptome. Our studies explore how RNA modification contribute to neurodegeneration, the functional consequences of RNA modifications in neurodegenerative pathways, and the potential of targeting the epitranscriptome for therapeutic development.

## 2 PATHOPHYSIOLOGY OF NEURODEGENERATION

Neurodegenerative disorders represent a group of pathological conditions characterized by the gradual and irreversible dysfunction and depletion of neurons and synapses in specific nervous system regions.^40^ This ultimately dictates the clinical manifestations and disease progression.^40^, ^41^ Generally, neurodegenerative disorders are associated with the buildup of certain proteins, the gradual decline of nerve cells, neuronal cell death, and susceptibility to specific brain regions.^42^ Prominent pathological characteristics in the development and progression of neurodegenerative disorders include persistent inflammatory responses and indications of immune activation within the CNS.^43^ Iron exhibits a range of both pro‐inflammatory and anti‐inflammatory activities. Improper regulation of iron chelation could potentially play a significant role in the development of inflammatory conditions and neurodegenerative disorders.^44^ The involvement of innate “protective autoimmunity” and the reparative functions of autoimmunity in neurodegenerative disorders is a significant aspect to consider.^45^ Disruption of the BBB allows blood‐borne molecules, especially plasma proteins, to enter the brain. This has been suggested as an initiating mechanism for certain neurodegenerative disorders.^43^ In an AD brain with heightened inflammation, a compromised BBB leads to the infiltration of fibrinogen and boosts microglial activity. These factors have been observed to collectively contribute to neuronal damage.^46^ Additionally, in PD, the neurotoxin 1‐methyl‐4‐phenyl‐1,2,3,6‐tetrahydropyridine (MPTP), has been demonstrated to cross the BBB. Once it is metabolized, it forms a toxic substance that could infiltrate and damage dopaminergic neurons mimicking the primary behavioral and pathological characteristics of PD.^47^, ^48^

Reactive oxygen species (ROS) could potentially play a pivotal role as a fundamental mechanism impacting the outlook of neurodegenerative disorders. Mitochondria serve as both targets and significant sources of ROS. Neural system (NS) mitochondria exhibit heterogeneity and variations in their functioning due to regionally specific regulatory factors and their energy production tailored to local requirements. Consequently, due to this heterogeneity, mitochondria display selective susceptibility to damage.^49^ Elevated levels of oxidants can trigger the mitochondrial permeability transition, disrupting oxidative phosphorylation. This disruption can lead to compromised energy production by mitochondria and can result in significant consequences. This can lead to cytotoxicity through processes like necrosis and apoptosis. In neurodegenerative disorders, there exists a bidirectional interplay involving mitochondrial fusion, fission, transport, and mitophagy.^50^ Dysfunctional bioenergetics and disruptions in mitochondrial energy metabolism result in decreased ATP generation, compromised calcium regulation, and the production of ROS. This combination is often described as a “deadly triad” in neurodegenerative disorders.^51^

Cell death in neurons is a significant feature and a crucial risk factor influencing the outcome of diverse neurodegenerative disorders. Several inherent characteristics of neurons may render them particularly susceptible to cell death in neurodegenerative disorders. This is probably due to the reason that neurons do not undergo cell division, leading to the gradual accumulation of age‐related damage to DNA, lipids, proteins, and organelles. Neurons have substantial energy requirements, primarily to support synaptic function. This results in the production of ROS through mitochondrial oxidative phosphorylation. This process can lead to brain volume reduction, which can be observed through volumetric magnetic resonance imaging (MRI), and the release of intraneuronal proteins like tau and neurofilaments into biofluids.^52^, ^53^ Excitotoxicity can also lead to neuronal demise by causing an excess of cytoplasmic calcium, which triggers a range of neuronal death programs.^52^ Neurons are among the most active and energy‐intensive cells in the human body. Impairments in energy metabolism have been demonstrated to play a role in a wide range of neurodegenerative disorders.^54^, ^55^ In various neurodegenerative disorders like AD and PD, neuronal cell death can occur through a distinct mechanism, specifically triggered by energy depletion.^56^ In many neurodegenerative disorders, synaptic dysfunction and toxicity appear to be an early occurrence that precedes the loss of neurons.^57^, ^58^ The proper functioning of neuronal networks relies on the precise operation of synapses, along with the regulated control of both synapse stability and removal.

**Figure 1:**
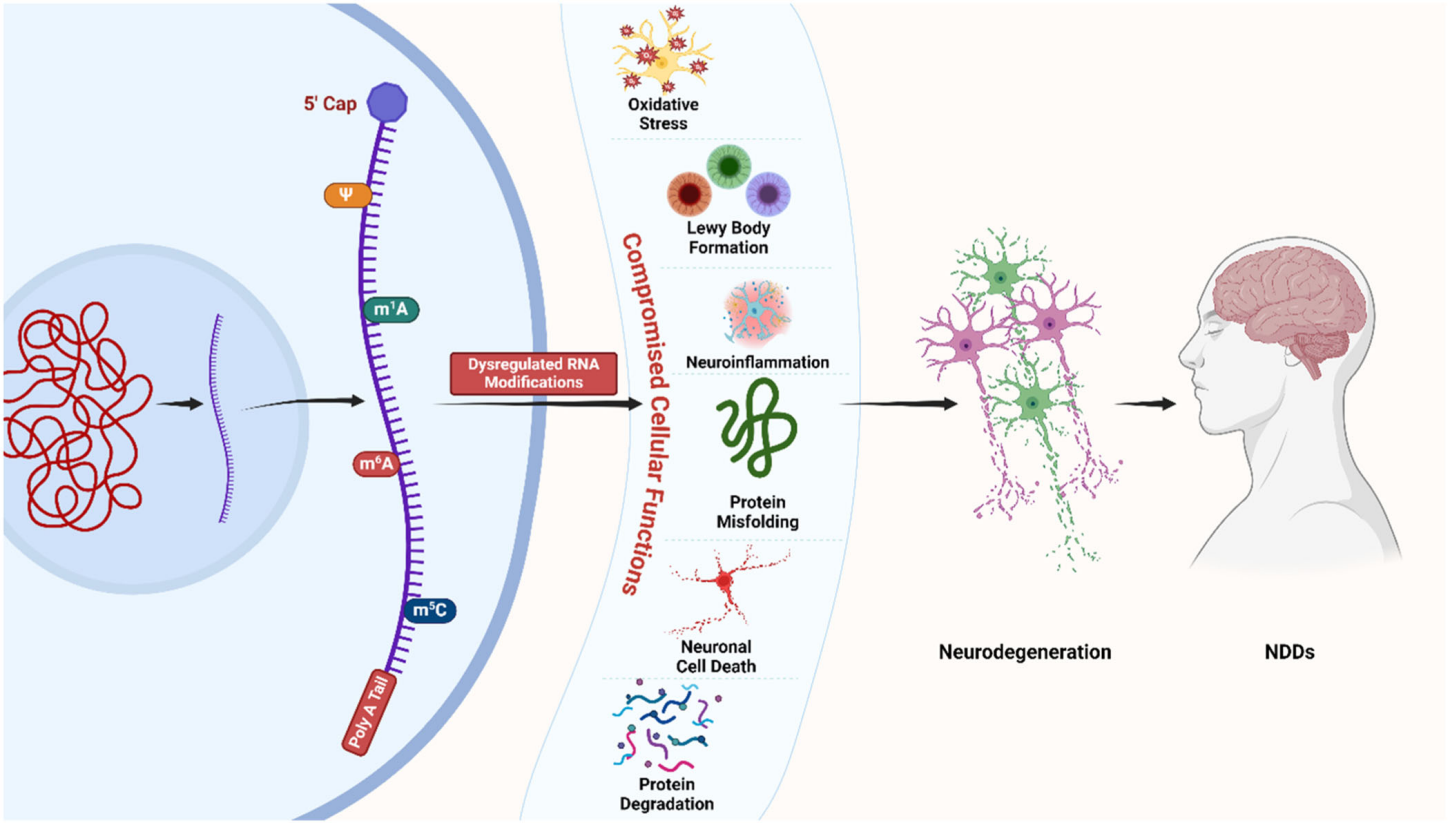
Mechanism of RNA modifications in the neurodegeneration and etiology of various neurodegenerative disorders. This shows a schematic pathway from RNA modifications leading to various neurodegenerative disorders. After an RNA gets transcribed, and in case of dysregulated RNA modifications as depicted, brain cells and tissues come under various cellular stresses including oxidative stress, Lewy body formation, neuroinflammation, protein degradation, neuronal cell death, and protein misfolding. These stresses ultimately result in the death of brain cells and tissues, eventually leading to the neurodegeneration and neurodegenerative disorders. NDDs, neurodegenerative disorders. [Color figure can be viewed at wileyonlinelibrary.com]

Neurodegeneration poses significant challenges, with aging populations contributing to their increasing prevalence. While the exact causes of most cases remain elusive, our understanding of the underlying mechanisms has grown substantially. In recent advancements, epitranscriptomics has shown significant promise in understanding and potentially treating neurodegenerative disorders. Consistent with important functions in neural development,^59^, ^60^ neurogenesis,^61^ learning and memory,^62^ and stress response,^63^, ^64^ epitranscriptomics provides a fresh perspective on pathogenesis of neurodegenerative disorders (Figure 1), offering hope for more effective treatments and potential cures. Current evidence suggests that various modifications of RNA such as m6A are implicated in various neurodegenerative disorders, including AD, PD, schizophrenia, and attention‐deficit/hyperactivity disorder (ADHD). This involvement is mediated through the regulation of gene expression and RNA metabolism.^65^, ^66^ By targeting and modulating these RNA modifications, it may be possible to develop novel therapeutic strategies for neurodegenerative disorders. Identifying potential risk factors and developing better animal models are essential steps to reduce the burden of these diseases and advance the quest for effective treatments.^67^

## 3 RNA MODIFICATIONS AND REGULATION OF GENE EXPRESSION

The conventional representation of RNA structure, being comprised of merely four A, C, G, and U nucleotides, has been redefined due to the discovery of modifications within these elemental components.^68^ Epitranscriptomic regulators, such as RNA modifications, play an essential role in gene expression control. RNA modifications are biochemical changes that occur in RNA molecules.^69^, ^70^, ^71^ The majority of these alterations are recognized for their dynamic and reversible properties, which contribute considerably to posttranscriptional gene expression control.^68^ RNA modifications, as reported by several recent studies, are crucial for mediating the process of gene expression as these epitranscriptomic factors have been shown to affect different aspects of RNA metabolism including transcription, pre‐mRNA splicing, exporting of mRNA, and the process of translation, subcellular localization, stability, and degradation.^16^, ^69^, ^70^, ^72^, ^73^, ^74^ These specific molecular processes maintain the stability of the mRNA molecule, which is crucial to gene expression.^74^ When an RNA molecule gets transcribed, specific writer enzymes install these alterations, which are then identified by reader proteins and, in some cases, erased.^15^ Modifications to pre‐mRNA during transcription may affect the binding of splicing factors, regulating alternative splicing and producing a variety of mRNA isoforms.^75^ This influences the sorts of proteins that may be produced from a single gene, which contributes to cellular variety and function.^76^ Modifications to mRNA may impact its export from the nucleus to the cytoplasm.^16^ Modified mRNAs are often identified by export receptors, allowing for efficient transit via the nuclear pore complex. In the cytoplasm, these changes are crucial in influencing mRNA stability and translation efficiency.^74^ For example, reader proteins may attract translation initiation factors to changed mRNAs, increasing ribosome recruitment and boosting effective translation.^70^ Furthermore, RNA modifications may affect the interaction of mRNAs with miRNAs or RNA‐binding proteins, hence regulating mRNA stability and translation.^74^ Specific changes may identify mRNAs for degradation by directing them to cellular structures like P‐bodies, where they undergo decay mechanisms.^77^ This targeted degradation ensures that gene expression levels remain carefully regulated, avoiding abnormal protein creation.^78^ Overall, RNA modifications act as dynamic regulators of gene expression, fine‐tuning cellular responses to developmental and external stimuli. These alterations, which influence various stages of the RNA life cycle, help to regulate gene expression precisely, which is required for maintaining cellular homeostasis and function.

**Figure 2:**
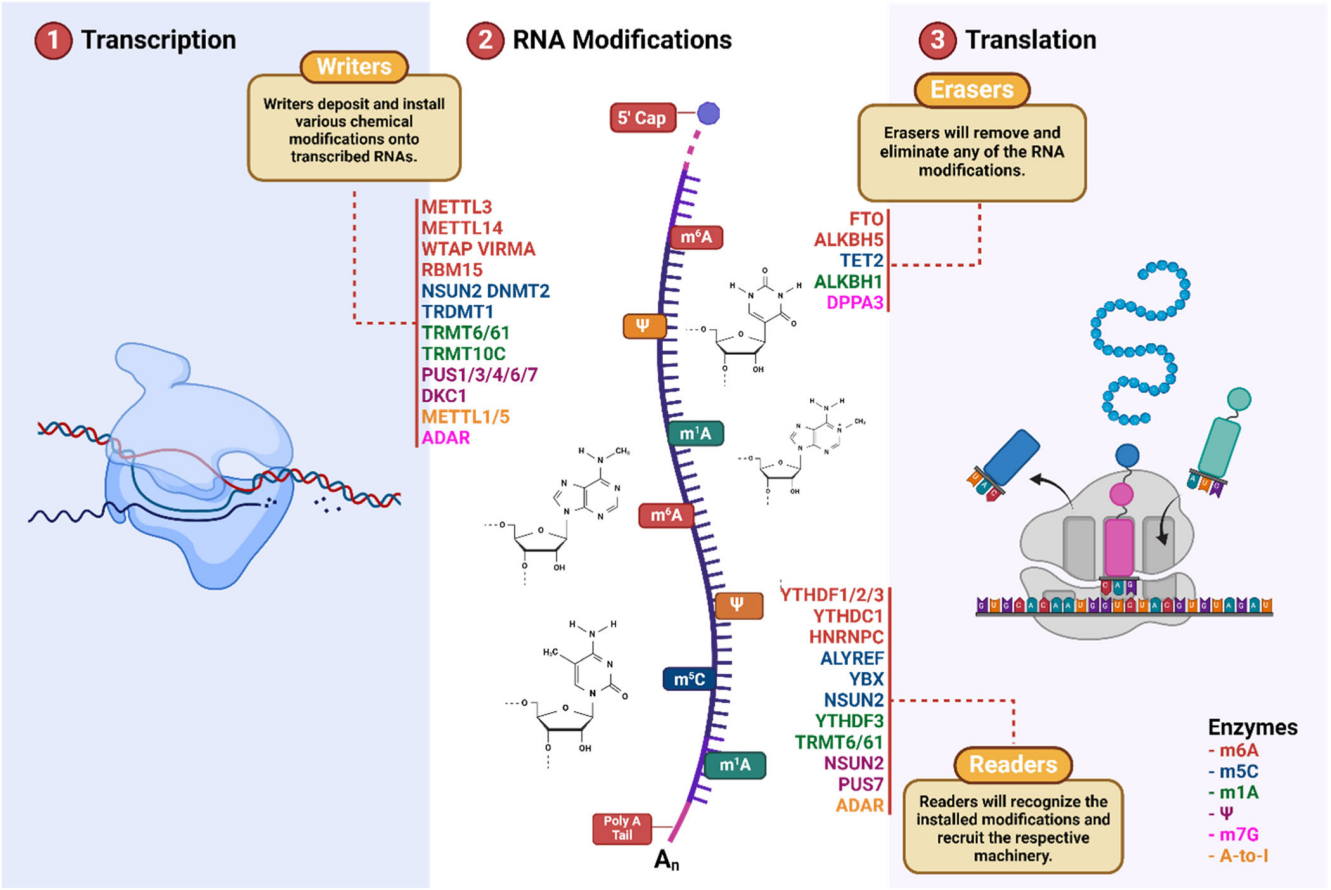
Role of RNA modifications and related enzymes in regulating gene expression. After RNA is transcribed, “Writers” deposit and install various chemical modifications onto the transcribed RNAs. “Erasers” are the enzymes which remove and eliminate any of the RNA modifications. During the final stage, the modified RNA is translated into a protein by the ribosome. These modifications are recognized and worked upon by the enzymes called “Readers” which then recruit respective machinery during translation. Key enzymes include METTL3/METTL14 (for m6A), NSUN2 (for m5C), TRMT6/61 (for m1A), PUS1 (for Ψ), ADAR (for A‐to‐I), YTHDF1/2/3 (for m6A), HNRNPC (for m6A), FTO (for m6A), and ALKBH5 (for m6A). Different colors represent corresponding RNA modifications. Fat mass and obesity‐associated protein (FTO); m6A, N6‐methyladenine; m5C, 5‐methylcytosine; m1A, N1‐methyladenine; Ψ, pseudouridine; A‐to‐I, adenosine‐to‐inosine. [Color figure can be viewed at wileyonlinelibrary.com]

To date, more than 170 RNA modifications have been reported, such as m6A, N6,2′‐O‐dimethyl adenosine (m6Am), 8‐oxo‐7,8‐dihydroguanosine (8‐oxoG), pseudouridine, m5C, and N4‐acetylcysteine (ac4C), have been shown to regulate mRNA stability, thereby playing an essential role in the gene expression (Figure 2).^79^, ^80^, ^81^ RNA modifications play an important role in the precise control of gene expression within neurons and the nervous system as well.^82^ Emerging research has shed light on the dynamic nature of RNA modifications, such as m5C and m6A, in neuronal responses to environmental signals like depolarization. These modifications have a strong link to the regulation of gene expression and are especially enriched in genes that govern critical neuronal functions.^83^ Due to the importance of these RNA modifications, their detection during a study becomes vital. Scientists use a plethora of techniques to detect these modifications.^84^ Techniques including liquid chromatography‐tandem mass spectrometry (LC‐MS/MS),^85^ high‐throughput sequencing methods like m6A‐RNA‐sequence technique (m6A‐seq) and methylated RNA immunoprecipitation sequencing (MeRIP‐seq),^86^, ^87^ m6A selective allyl chemical labeling and sequencing (m6A‐SAC‐seq),^88^ and nanopore sequencing have proven effective for detecting and quantifying RNA modifications.^86^ Additionally, machine learning models trained on synthetic controls are used to assess modifications such as pseudouridine occupancy in mRNAs, enabling precise evaluation of these modifications.^89^

## 4 M6A

m6A is a biochemical alteration of mRNA that seems to exist in eukaryotic cells.^90^, ^91^ It is likely the most common and prevalent of all RNA modifications.^90^ It has been shown to play a part in several biological functions, such as cell division, apoptosis, and self‐renewal. It has also been linked to the onset and development of a variety of illnesses, such as autoimmune diseases, gliomas, carcinogenesis, cardiovascular diseases, and neurological disorders.^92^, ^93^, ^94^, ^95^, ^96^, ^97^ The m6A alteration, for example, is important in modulating neurogenesis in the midbrain of mammals, brain development in the embryo, and CNS growth.^94^ The effects of the m6A modification on various pathophysiological and physiological functions are evident from its ability to regulate gene expression.^91^ The RNA m6A machinery, which consists of writers, erasers, and readers of m6A, is necessary for the deposition and functioning of m6A.^98^ Methyltransferases, usually referred to as writers, such as methyltransferase 3, N6‐adenosine‐methyltransferase complex catalytic subunit (METTL3), methyltransferase 14, N6‐adenosine‐methyltransferase complex catalytic subunit (METTL14), Vir like M6A methyltransferase associated (VIRMA/KIAA1429), RNA binding motif protein 15 (RBM15/15B), zinc finger CCCH‐type containing 13 (ZC3H13), and WT1 associated protein (WTAP), most frequently add m6A to mRNA. M6A is eliminated from mRNA by demethylases, called erasers such as AlkB homolog 5 (ALKBH5) and fat mass and obesity‐associated protein (FTO).^99^, ^100^ The third type of RNA‐modifying proteins called readers also exert biological effects of m6A on target mRNAs, including the YTH domain family, heterogeneous nuclear riboproteins (HNRNPs), GF2BP1‐3, eukaryotic translation initiation factor 3 (EIF3), and insulin like growth factor 2 mRNA binding protein (IGF2BPs).^101^, ^102^

Recent research has shown that pleiotropic factors can stimulate the m6A machinery to chromatin. Therefore, in addition to installing m6A on nascent mRNA, the recruitment of m6A machinery can also have a direct impact on chromatin biology, including transcription, and posttranscriptional modifications.^98^ Due to the remarkable ability of m6A modification to directly affect and regulate the biology of chromatin and DNA, abnormal m6A modification functioning has been associated with neurodegenerative illnesses and damage to the brain.^103^ In neurobiology, m6A contributes to neurodegeneration, brain plasticity, and neural development.^65^ Furthermore, m6A modifications exert a significant influence on various aspects of neuronal function and brain development, including dendritic structure, spinogenesis, learning and memory processes, neurogenesis, axon regeneration, and overall brain development.^104^ Neurological diseases result from disrupted m6A pathways, often due to disease‐specific mutations or fluctuations in m6A‐related components, though the precise mechanisms are not fully comprehended.^105^ Many recent studies have demonstrated that Lewy body illnesses, Huntington's disease (HD), and the aging mouse hippocampus are affected by the expression levels of m6A modification in the brain.^106^, ^107^, ^108^ Furthermore, many other studies have also reported that m6A dysregulation has been associated with AD, PD, Fragile X syndrome, HD, and intellectual disability.^99^, ^109^, ^110^, ^111^ In another study, m6A methylations have been discovered as possible biomarkers in cognitive dysfunctions.^112^

Similarly, abnormal m6A modifications have also been observed to influence compromised nervous system activities, including retardations in the development and differentiation of brain tissues, disorders of axon regeneration, memory issues, and neuronal cell regeneration and differentiation disorders.^113^, ^114^ According to research, a significant number of m6A transcripts are localized at synapses, involved in the control of synaptic processes, and conserved there.^115^ m6A alteration is also linked with the differences in synaptogenesis which is also a risk factor for various neurodegenerative disorders.^109^, ^116^, ^117^ Reduced m6A RNA methylation has been detected in both human brain tissue from the cingulate gyri (arch‐shaped convolution situated just above the corpus callosum) of AD patients and brain tissue from a mouse model of AD.^115^ Enlargement of the lateral ventricular region of the brain has been observed to be associated with several neurodegenerative disorders like AD and PD. A genome‐wide association study (GWAS) revealed a significant relationship between the abnormal function of the m6A RNA modification and the enlargement of the lateral ventricles in the brain, indicating its involvement in neurodegenerative disorders.^118^ m6A modification has also been observed to be involved in neuronal cell death by several mechanisms. For instance, in a study, m6A methylation of the long noncoding RNA Lnc‐D63785 triggered by oxygen glucose deprivation/reoxygenation (OGD/R) led to an accumulation of microRNA miR‐422a, resulting in the downregulation of key neuronal survival factors, myocyte enhancer factor 2D (MEF2D) and mitogen‐activated protein kinase kinase 6 (MAPKK6), ultimately causing neuronal apoptosis.^119^ Neuronal cell death is a major characteristic feature of many neurodegenerative disorders such as AD, ALS, HD, and PD.^120^

In order the understand the involvement of m6A in neurodegenerative disorders, a study focused on a double transgenic mouse model (APPNL‐G‐F/MAPTP301S) that exhibits major AD pathologies, including beta‐amyloid peptides (Aβ) plaque accumulation, tau pathology, inflammation, and neurodegeneration. This model demonstrated significant m6A accumulation in neurons and glial cells, along with increased METTL3 and decreased ALKBH5 levels. The findings suggest that m6A modifications play a crucial role in AD‐related neurodegeneration, as the mouse model recapitulates many features of the disease.^121^ Research on human AD brains further explored the role of m6A modification and the expression of m6A regulators. Reduced m6A levels and METTL3 expression were observed in AD brains, leading to memory deficits and neuronal death in models with METTL3 knockdown. This study concluded that METTL3 reduction‐mediated m6A dysregulation significantly contributes to AD neurodegeneration and presents a potential therapeutic target.^122^ Additional research analyzed the temporal and spatial dynamics of m6A modification during neurodevelopment and aging. It was found that m6A levels increase with age, affecting RNA metabolism and expression in a tissue specific manner. In AD models, decreased m6A on AD‐related transcripts correlated with reduced protein levels. These findings suggest that m6A exerts a critical function in brain development and aging, impacting key genes involved in AD.^123^

Similar studies have been conducted to elucidate the relationship of m6A with other neurogenerative disorders. A 2023 study on HD found that aberrant splicing in HD was linked to disrupted TAR DNA binding protein (TDP‐43) activity and altered m6A RNA modification. TDP‐43 and the m6A writer protein METTL3 were identified as regulators of exon skipping in HD. The study suggests that dysregulated m6A modification, mediated by TDP‐43 and METTL3, contributes to HD pathogenesis by affecting RNA splicing. Targeting m6A pathways may offer new therapeutic approaches for HD, providing insights into its molecular mechanisms.^124^ Similarly, a research on m6A modification in PD examined m6A levels and modulators in peripheral blood mononuclear cells. The findings revealed substantially reduced mRNA levels of m6A, METTL3, METTL14, and YTH N6‐methyladenosine RNA binding protein F2 (YTHDF2) in PD patients compared to controls, with METTL14 identified as the primary cause. Mechanistic investigations demonstrated that METTL14 controls α‐synuclein (α‐syn) gene expression via m6A modification, influencing α‐syn mRNA stability. Overexpression of METTL14 led to increased m6A modification of α‐syn mRNA, which decreased its stability. The research indicated that METTL14 might serve as a potential diagnostic biomarker for PD and found a m6A‐YTHDF2‐dependent pathway for regulating pathogenic α‐syn proteins.^125^ Collectively, these studies demonstrate that dysregulation of m6A methylation plays a significant role in the development and progression of neurodegenerative disorders, underscoring its potential as a therapeutic target.

## 5 M5C

The m5Cmodification involves the addition of a methyl group to the fifth carbon of cytosine in RNA molecules.^126^ This common modification has been observed in mRNA, tRNA, rRNA, long noncoding RNA (lncRNA), small nuclear RNA (snRNA), miRNA, and enhancer RNA (eRNA).^126^, ^127^ TRNA aspartic acid methyltransferase 1 (DNMT2) and the NOP2/Sun RNA methyltransferase (NSUN) are the most importantly recognized methyltransferase that deposits the m5C modification on RNA.^101^ Readers of m5C, consist of ALYFREF and Y‐box binding protein 1 (YBX1). The TET family of enzymes has been proposed to potentially function as m5C erasers.^128^ It has been observed that m5C modification regulates a variety of physiological and complex cellular processes, including RNA metabolism, RNA stability control, gene expression, cellular development, cell migration, and transcriptional regulation.^126^, ^129^ Furthermore, m5C modification can impact the translation process by affecting aminoacylation, potentially altering translational accuracy.^104^ Recent investigations involving *Arabidopsis thaliana* and zebrafish have unveiled the regulatory role of m5C modification in mRNA stability.^104^ In HeLa cell lines, it was observed that NSUN2‐mediated m5C modification of mRNAs facilitates their interaction with ALYREF, an mRNA transport adaptor crucial for nuclear export.^130^ It has been observed that mutated RNA m5C methyltransferases and altered m5C modifications are associated with several disorders which may include disorders of stem cells, cancer, developmental disorders, and neurodegenerative disorders.^131^, ^132^, ^133^

In the brain and nervous system, the distribution of m5C modifications is substantially conserved, greatly enriched in regions rich in CG content, and concentrated in the regions where translation initiates.^132^, ^134^, ^135^ m5C modification has been observed in the mouse brain cells indicating its potential role in the neuronal functions.^131^ By suppressing the production of neuronal mRNAs, m5C modification has been observed to disrupt signaling pathways in the development of neurons which is a hallmark of neurodegenerative disorders.^82^ In 2022, a study demonstrated direct relation between the m5C and neurodegenerative disorders. A study found that the m5C methyltransferase NSUN2 is downregulated in the brains of AD patients, resulting in decreased m5C levels in tRNA. This reduction impairs tRNA stability and function, contributing to AD pathogenesis. Overexpression of NSUN2 in a mouse model of AD ameliorated cognitive deficits and reduced neurodegeneration, suggesting therapeutic potential.^136^ Additionally, m5C modifications have been linked to the inverse comorbidity between cancer and neurodegenerative disorders. Certain m5C‐related genes, such as DNA methyltransferase 3 alpha (DNMT3A) and Tet methylcytosine dioxygenase 2 (TET2), frequently mutated in myeloid malignancies and are also associated with an increased risk of neurodegenerative disorders. The same m5C‐related noncoding RNAs can contribute to the pathogenesis of both cancer and neurodegeneration, depending on the specific cellular context and signaling pathways activated.^137^ Furthermore, disturbances in m5C‐related enzymes and receptors can disrupt gene expression, protein equilibrium, and neuronal function, thus contributing to neurodegeneration. A deeper exploration is imperative to comprehend the intricate mechanisms through which m5C modifications affect neurodegeneration and to devise targeted therapeutic interventions centered on m5C regulation.^138^

## 6 M1A

m1A is a modification that happens when a methyl group is added to the nitrogen atom at position 1 of the adenine base in RNA. This modification is installed on RNA by TRNA Methyltransferase (TRMT)‐10C (TRMT10C), TRMT6, TRMT61A, and TRMT61B complex.^101^, ^128^ m1A erasers include ALKBH1 and ALKBH3.^128^ m1A has been observed to be linked with the regulation of gene expression and cellular processes.^139^ It has also been demonstrated that m1A influences tRNA function and processing, miRNA processing, and mRNA stability. Its particular mechanisms and functions are yet unknown and are being explored.^140^ Previous research has demonstrated that m1A, which is found in tRNA and rRNA and modifies the structural stability of RNA by changing its secondary structure, may influence protein translation efficiency.^141^, ^142^ This notion was substantiated by more recent research that indicated that m1A is also common in the mRNA of eukaryotic cells and that it can enhance protein translation since it is abundant in the mRNA's 5' UTR andproximity to the initiation codon.^143^, ^144^ The emergence of m1A as a noteworthy RNA modification has sparked growing interest in its potential implications within the landscape of neurodegenerative disorders. Initially recognized for its role in mRNA and tRNA modification, recent research has unveiled the significance of m1A modification within the intricate milieu of the CNS.^30^, ^145^, ^146^ The dynamic relationship between m1A modification and RNA metabolism within neurons introduces a fresh perspective on the complex interplay between RNA regulation and disease development.^30^, ^144^, ^147^ The role of m1A in modulating RNA stability and translation efficiency adds an additional layer of complexity to our understanding of neurodegenerative processes.^147^, ^148^ m1A plays crucial roles in RNA metabolism and biological processes. In a study using the m1A‐quant‐seq approach, hypomethylation of mitochondrial and cytosolic tRNAs was observed in an AD mouse model (5XFAD). Enzymes responsible for m1A addition displayed decreased expression, correlating with altered mature mitochondrial tRNA levels and exacerbating tau pathology in a Drosophila model.^149^ Investigating the m1A modification's role in stress granule formation, another study utilized a *Caenorhabditis elegans* model of PD. It revealed that m1A modification contributed to CAG repeat expansion‐induced neurodegeneration by enhancing TDP‐43's partitioning intostress granules. This implicates m1A modification in regulating TDP‐43 activity, suggesting its involvement in PD pathogenesis.^150^ These findings suggest a potential role of hypo m1A modification in tRNAs in AD pathogenesis.

So far, there is no clear link established between the m1A modification and the pathophysiology of neurodegeneration due to the lack of prominent studies, but provided that more research is carried out on this venture, can provide deep insights due to the involvement of this modification in the functions of the neurons and nervous system.

## 7 PSEUDOURIDINE MODIFICATION

One of the most prevalent Posttranscriptional RNA alterations that is actively distributed across the transcriptome is pseudouridine. It is usually pervasive in multiple RNAs. It may have an impact on several facets of RNA biology, giving the RNA molecules with their unique structural and functional characteristics.^151^ Pseudouridine is created by isomerizing uridine by rupturing the glycosidic link, rotating the base 180 degrees, and re‐forming the bond.^152^ There are two classes of pseudouridine synthases (PUSs), and their main purpose is the guidance of pseudouridylation.^153^ One type is the RNA‐dependent PUSs. These PUSs in yeast are known as Cbf5 and in humans, they exist as dyskerin, which interacts with the box H/ACA small nucleolar ribonucleoproteins (snoRNPs), and after target recognition by base pairing of snoRNA and the substrate RNA, rRNA structural conformation is primarily altered by directly recognizing the sequence and structural facets inside these molecules.^152^ The RNA‐independent PUS proteins target site‐specific alteration of tRNA, short nuclear RNA, and rRNA. The majority of the enzymes involved in altering eukaryotic noncoding RNAs have been found through genetic study in budding yeast and have been extended to human cells through homology.^153^

An important question is how these mRNA enzymes identify these modifying enzymes of tRNA. Sequence patterns discovered in mRNA focus on genetically assigned TruB pseudouridine synthase family member 1 (PUS4) and pseudouridine synthase 7 (PUS7) with matching motifs which have been known to be important and dire for these changes of tRNA, inferring that mRNA and tRNAs have some comparable ways of recognizing and binding to the enzymes responsible for their modifications.^154^, ^155^ In short, the pseudouridine modifications alter the secondary structure by the addition of more base pairs or making the structure more rigid especially the backbone of the molecule, and also by adding various ridges and through base pair stacking. Pseudouridine synthase mutations have been linked to mitochondrial myopathy and sideroblastic anemia (MLASA), dyskeratosis congenita, and lung cancer. But these are not the only diseases, it has been noticed that this molecule or pseudouridine synthase has actively participated in neurogenerative disorders. The basic mechanism that links to neurodegenerative anomalies often results from alternative mRNA splicing. Numerous investigations have shed light on how pseudouridylation affects neuronal operations. For instance, the deletion of the Drosophila PUS enzymes RluA‐1 and RluA‐2, which are unique to nociceptor neurons, results in hypersensitive nociception symptoms such as heat hyperalgesia.^156^ Levels are elevated in the urine of AD patients, but no apparent symptoms have been directly related to this characteristic.^157^ Muscleblind‐like 1 protein no longer binds to CCUG repeat‐expanded cellular nucleic acid‐binding protein (CNBP) RNA connected to type 2 myotonic dystrophy (DM2) pathogenicity as a result of modification of the enlarged intronic CCUG repeats in CNBP, whose expression is linked to DM2 in humans.^158^ This shows that pseudouridine could be used as a target of therapeutic potential.

## 8 A‐TO‐I EDITING

Adenosine deamination in the transcriptome produces inosine, which is known as A‐to‐I RNA editing. Adenosine deaminase catalyzes A‐to‐I editing by acting on adenosine deaminase RNA specific (ADAR) enzymes.^159^ Through the hydrolytic deamination of the amino group at the C6 position of adenosine, adenosine is transformed into inosine. A Posttranscriptional mechanism for enhancing the proteome repertoire is A‐to‐I RNA editing by ADARs. Posttranscriptional RNA modifications of many different kinds are regularly applied to eukaryotic RNA transcripts, which helps to regulate gene expression in a variety of biological processes. Growing evidence suggests that these posttranscriptional RNA changes are crucial to the CNS's complex functioning.^160^ The development of a double‐stranded RNA (dsRNA) structure in the nucleus is necessary for this reaction. Inosine and guanosine have a chemical structure, therefore instead of pairing with uridine, inosine bases do. During the splicing of mRNA along with translation for protein synthesis, the inosines of the RNA transcripts convert to guanosines after translation. A‐to‐I RNA editing, also characterized as A‐to‐I modification, is the consequence of this editing of genomic sequences.^161^

In metazoans, the ADAR gene family is highly conserved. There are now three members of the ADAR gene family known to exist in vertebrates: ADAR1, ADAR2, and ADAR3. They share similar domain elaborations. They have several tandemly repeated double‐stranded RNA binding domains (dsRBD) in the N‐terminal region that allow for direct binding with dsRNA structures.^162^ It is well known that ADAR1 is principally in charge of RNA editing in repeat motifs of mRNAs' non‐'coding regions. Though most protein‐coding genes are expressed in the CNS, ADAR2 is primarily involved in recording editing in these genes. More A‐to‐I RNA editing sites can be found in noncoding than coding regions of mRNAs. It has been claimed that some of them play a role in controlling how genes are expressed. Splicing sites are created or eliminated by RNA editing in the intronic sections of mRNAs to control alternative mRNA splicing.^163^, ^164^

According to a current study, ADAR2‐dependent A‐to‐I editing controls the cyclical oscillation of mRNA expression levels during the circadian cycle.^165^ It is known that ADAR1 mutations are linked to bilateral striatal necrosis (BSN), an inflexible movement disease resulting from brain anomalies. Interferon signals are a characteristic of the BSN resulting from ADAR1 mutations.^166^ A chronic lymphocytosis in the cerebral fluid characterizes the deadly childhood encephalopathy known as Aicardi‐Goutières syndrome (AGS). Ten ADAR1 mutations have been recognized as probable AGS causes to date.^167^ Adenosine deaminases acting on ADARs catalyze A‐to‐I RNA editing, which modifies RNA transcripts to promote genomic diversity.^168^ This mechanism is crucial for CNS development and function, and dysfunctions in ADARs may lead to neurodegenerative, mental illnesses, as well as CNS malignancies.^169^, ^170^ ADARs target major components of the CNS, such as glutamate receptors, serotonin receptors, and potassium channels.^171^ They interact with hundreds of genes and millions of editing sites which make this modification and important agent in neurodegeneration.^172^ In AD, a detailed analysis using advanced sequencing techniques found that RNA editing levels are reduced in brain tissues, especially in the hippocampus, and to a lesser extent in the temporal and frontal lobes. This study identified 35 sites within 22 genes with differential editing, suggesting a link between AD and impaired RNA editing.^173^ Another study focused on glutamate ionotropic receptor AMPA type subunit 2 (GluR‐2) RNA editing, which affects calcium permeability in neurons, revealing that in the prefrontal cortex of Alzheimer's and schizophrenia patients, approximately 1.0% of GluR‐2 RNA remained unedited compared to less than 0.1% in controls. In HD, about 5.0% of GluR‐2 RNA in the striatum was unedited. These findings indicate that disrupted GluR‐2 RNA editing, leading to increased calcium permeability, may contribute to neuronal dysfunction in schizophrenia and neuronal death in AD and HD.^174^ This underscores the importance of RNA editing in maintaining neuronal function and its role in the pathogenesis of neurodegenerative disorders.

## 9 RNA‐MODIFYING PROTEINS AND THEIR ROLE IN NEURODEGENERATION

**Figure 3:**
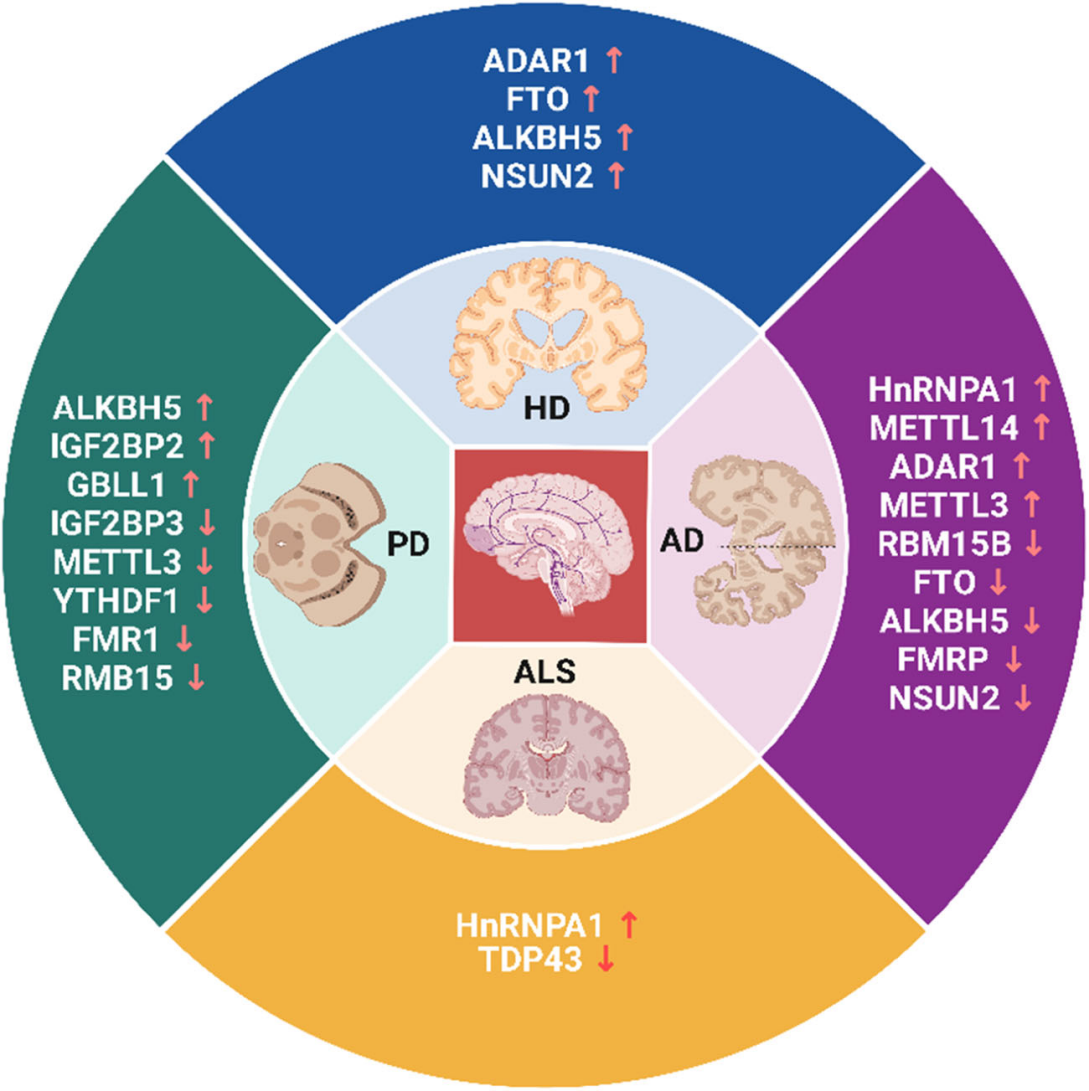
Regulation of different RNA‐modifying proteins in neurodegenerative disorders. *↑ refers to upregulation and ↓ refers to downregulation of each enzyme. AD, Alzheimer's disease; ALS, amyotrophic lateral sclerosis; HD, Huntington's disease; PD, Parkinson's disease. [Color figure can be viewed at wileyonlinelibrary.com]

Recently, RNA modifications have been observed as important posttranscriptional regulators of gene expression programs, and many of these changes must be correctly deposited for proper development.^133^ In addition to the four basic A, U, C, and G nucleotide bases, RNA molecules may also contain any of the 170+ recognized biochemically modified residues.^175^, ^176^ Almost all of these naturally occurring RNA modifications are induced by the enzymes known as RNA‐modifying proteins.^177^ RNA‐modifying proteins are the proteins that recognize, induce, and erase RNA modification. Writers and erasers are the enzymes that install and remove these modifications in a site‐specific manner whereas reader proteins may interpret alterations and transduce the signal for downstream actions.^178^ Currently, there are over 350 recognized RNA‐modifying proteins and the number is on a constant rise.^15^ These enzymes are grouped into three categories: RNA modification deposition effectors (writers), RNA modification recognition effectors (readers), and RNA modification removal effectors (erasers).^179^ These RNA‐modifying enzymes have been seen to play a very crucial role in several disorders including neurodegenerative disorders (Figure 3).^15^ RNA modifications are known to influence gene expression, and new data shows that they may also play a role in the DNA damage response (DDR) pathways, which are critical for genome integrity. Changes in RNA have a significant impact on DNA‐RNA hybrids (R‐loops) at DNA damage sites, a structure that is known to be critical in the repair of DNA and the integrity of the genome.^180^ These RNA‐DNA hybrids emerge when RNA transcripts originating from regions undergoing active transcription adhere to DNA double‐strand breaks (DSBs), hindering the repair process through both homologous recombination (HR) and nonhomologous end joining (NHEJ) pathways.^181^, ^182^ RNA modifications can alter the structure of RNA, affecting its ability to interact with DNA, thereby promoting or inhibiting R‐loop formation.^183^, ^184^ RNA modifications can also attract proteins that recognize the modified RNA, facilitating the formation or stabilization of R‐loops by affecting the DNA strand or supporting the RNA‐DNA hybrid.^185^ Specific RNA modifications might target proteins to particular genomic regions to promote R‐loop formation there.^184^ Given the importance of the DDR in inhibiting mutations and human diseases such as neurodegeneration, RNA modification pathways may have a role in neurodegenerative disorders not only via their functions in gene expression but also through their ability to modify DNA repair processes and the stability of the genome.^180^

MPTP is a synthetic drug that has been extensively utilized in generating animal models of PD, especially mice models.^186^ It selectively targets the dopaminergic neurons and induce characteristic symptoms of PD such as slow muscle movements, muscle rigidity and tremors.^187^, ^188^ Given that the MPTP‐induced PD mouse model has many characteristics of human illness, it has a great value for studying the pathophysiology of neurodegenerative disorder.^189^ Neuroinflammation and oxidative stress are seen in both human and animal models due to the increasing death of dopaminergic neurons, the buildup of α‐synuclein, and the activation of microglia and astrocytes.^190^, ^191^ This makes the model a valuable resource for understanding the fundamental causes of PD. In one such MPTP‐induced PD mouse model, the expression of m6A regulatory proteins differed between the substantia nigra (SN) and striatum regions. In the SN, ALKBH5 and IGF2BP2 were overexpressed, while YTH N6‐methyladenosine RNA binding protein F1 (YTHDF1) and fragile X messenger ribonucleoprotein 1 (FMR1) were under‐expressed. In the striatum, FMR1 and Cbl proto‐oncogene like 1 (CBLL1) were elevated, while IGF2BP3, METTL3, and RBM15 were reduced. The mRNA expression of these genes partly mirrored the changes in their corresponding proteins. These findings suggest that m6A regulators may play a pivotal role in the development of PD.^105^ According to a study of PD patients, increased levels of m6A modification of α‐synuclein were observed, which eventually compromised the stability of α‐synuclein, an important protein implicated in PD. The study revealed that there was a stark difference in concentrations of various m6A regulators between the healthy controls and PD patients' brains, with controls having significantly lower amounts of METTL14. When METTL14 was overexpressed, α‐synuclein's m6A modification was increased, which reduced the protein's aggregation and neurotoxicity. Based on these results, targeting the α‐synuclein m6A mutation may be a viable treatment strategy for PD.^125^

Heterogeneous nuclear ribonucleoprotein A1 (HnRNP A1) is a highly conserved protein with a key role as an RNA‐binding factor. Its involvement areas across a diverse range of crucial physiological and metabolic functions, including transcription, pre‐mRNA splicing, mRNA transport, protein translation, microRNA processing, telomere maintenance, and regulation of mRNA stability.^192^, ^193^ The deregulation of HnRNP A1 has been revealed to be particularly influential in the etiology of neurodegenerative illnesses such as ALS, frontotemporal lobar degeneration, multiple sclerosis (MS), spinal muscular atrophy, AD, and HD.^192^ FTO is an eraser of the m6A modification and it facilitates the oxidative demethylation of several RNA types.^194^, ^195^ FTO is said to be significantly expressed in both adipose and brain tissues as it has been observed to be involved in brain development and function.^195^, ^196^ FTO may also be associated with the environmental associated neurodegeneration. In a relatively recent study, the researchers investigated the role of FTO enzymes in neurodegeneration caused by Cobalt exposure in human neuroglioma H4 cells. Cobalt chloride (CoCl2) exposure decreased FTO expression and increased oxidative stress, leading to altered m6A modification of apoptosis‐related genes and activation of apoptosis. These findings indicate that variations in the expression levels of FTO may be involved in regulating apoptosis in neurodegeneration pathogenesis induced by environmental toxicants.^197^ In various other in vivo and in vitro studies, FTO has also been observed to induce neurodegeneration along with inflammation in the retinal neurons by promoting endothelial cell‐microglia interactions.^198^ These findings iterate the fact the various RNA‐modifying proteins have significant contribution to the pathogenesis of various neurodegenerative disorders by regulating the expressions of the RNA modifications.

## 10 FUNCTIONAL CONSEQUENCES OF RNA MODIFICATIONS IN NEURODEGENERATIVE PATHWAYS

RNA modifications undergo dynamic regulation across various RNA species within cells and hold critical roles in fundamental biological processes, notably cellular senescence and aging.^199^ Dysregulation of RNA metabolism has emerged as a central factor in neurodegenerative disorders, impacting mRNA processing and noncoding RNA biogenesis.^200^ Processes like the mRNA and RNA stability are all regulated by the RNA modification. As a result, RNA modifications play a pivotal role in the initiation and progression of neurodegenerative disorders as summarized in Table 1.

**Table 1:** Various important neurodegenerative disorders and the dysregulated expression of different RNA modifications and their cellular consequences.

Sr.	Disease	Localization	Modification	Consequences	Disturbed cellular functions	Reference
1.	***Parkinson's disease (PD)***	Brain	Pseudouridine modification	Loss of PUS7 and the presence of pseudouridine modification have been found to be associated with PD.	RNA stability is compromised, along with interactions with RNA‐binding proteins.	[201, 202]
			A‐to‐I RNA editing	Modified A‐to‐I RNA editing patterns are observed in PD.	mRNA stability, splicing, and protein function are all affected adversely.	[201, 203]
2.	***Alzheimer's disease (AD)***	Brain	m6A	Elevated levels of m6A observed in AD.	Reduced mRNA stability, altered splicing, and impaired translation.	[104, 201]
3.	***Amyotrophic lateral sclerosis (ALS)***	Brain and spinal cord	m5C	Lowered levels of m5C detected in ALS.	Reduced stability and translation efficiency of mRNA.	[104, 201]
			m1A	Elevated levels of m1a detected in ALS.	Reduced mRNA stability and hindered translation.	[104, 201]
4.	***Huntington's disease (HD)***	Brain	Pseudouridine	The absence of PUS7 and the loss of Pseudouridine modification are associated with HD.	Decreased RNA stability and disruptions in interactions with RNA‐binding proteins.	[201, 202]
			m6A	Elevated levels of m6A identified in HD.	Reduced stability of mRNA, altered splicing, and impaired translation.	[104, 201]
			m5C	Lowered levels of m5C observed in HD.	Reduced stability of mRNA, disrupted splicing, and impaired translation.	[104, 201]
			A‐to‐I RNA editing	Modified A‐to‐I RNA editing patterns observed in HD.	Reduced mRNA stability, disrupted splicing, and compromised protein function.	[203, 204]

Abbreviations: A‐to‐I, adenosine‐to‐inosine; m1A, N1‐methyladenine; m6A, N6‐methyladenine; m5C, 5‐methylcytosine.

### 10.1 Implications in mRNA splicing

RNA splicing is a critical step in RNA maturation. RNA modifications play a key role in mRNA splicing, and aberrant mRNA has been related to several neurological disorders. RNA modification dysregulation induced by abnormal expression of or mutations in RNA modifiers can remodel the epitranscriptome and skew overall gene expression, resulting in impaired cellular activities.^205^ Numerous common neurodegenerative disorders, such as AD, PD, HD, and spinal muscular atrophy, involve some sort of splicing defects.^206^, ^207^ For instance, Tau mis‐splicing has been frequently detected in several tauopathies, including AD, resulting in isoform‐specific impairments in normal physiological function and improved recruitment of excessive tau isoforms into the pathological process.^208^ Another aspect of RNA splicing is alternative splicing. mRNA alternative splicing is essential for isoform diversity and gene expression control.^209^ Alternative splicing of mRNA is a fundamental step in posttranscriptional control of gene expression, allowing eukaryotic organisms with limited gene counts to significantly expand their functional proteome by generating distinct mRNA isoforms from a single primary transcript.^210^ Studies have indicated that RNA modifications play a significant role in the regulation of mRNA splicing events. The m6A modification is specifically recognized by a group of proteins known as the YTH domain‐containing family, which includes YTHDC1‐2 and YTHDF1‐3. Notably, among these proteins, YTHDC1 serves as a nuclear m6A reader and exerts a significant regulatory influence over various RNA‐related processes, particularly alternative splicing and broader RNA metabolic pathways.^211^ The m6A modification exhibited a dual role, both promoting and inhibiting an equal number of alternative splicing events within mouse cell lines. This observation underscores the regulatory potential of m6A in orchestrating alternative splicing processes.^212^ Also, human telomerase reverse transcriptase (hTERT) pre‐mRNA alternative splicing at the posttranscriptional level is one of the mechanisms for regulating telomerase activity, which is linked to cancer and aging‐related disorders.^213^ TDP‐43 is a complex heterogeneous ribonucleoprotein that plays essential roles in mRNA processing and stabilization, notably during splicing. TDP‐43 disruptions or genetic abnormalities have been associated with severe human neurodegenerative illnesses, most notably ALS and frontotemporal lobar degeneration (FTLD). This highlights the important role of TDP‐43 in the pathophysiology of these diseases.^214^

Splice variants produced by alternative splicing may encode distinct protein isoforms. Specific domains can be lost or gained by these isoforms, resulting in differences in their characteristics. As a result, RNA splicing is important in determining protein variety by creating distinct variants of the same protein with diverse functional characteristics.^215^ Many isoforms of different proteins, resulting from RNA splicing have also shown their prominence in the pathogenesis of several neurodegenerative disorders. Amyloid precursor protein (APP) is an important protein in the neurological system. In the brain, it has three major isoforms including 695, 751, and 770 amino acids, which are the consequence of alternative splicing of a single gene on chromosome 21. The accumulation and deposition of Aβ, a fundamental component of neurotic plaques, is a hallmark of AD and associated neurological disorders. This Aβ is produced through the proteolytic cleavage of APP. During its maturation, APP undergoes various posttranslational modifications, including N‐ and O‐glycosylation, which are hypothesized to impact its expression and secretion.^216^ Abnormal splicing of mRNA due to the impairments in the RNA modifications have a major role in the tau‐pathologies and can thus be targeted for treatment of neurodegeneration.

### 10.2 Translation control

The translation is a crucial process in central dogma. The proper translation process is important as the body's normal working requires proper proteins. RNA modifications, such as those in tRNAs, play an important role in regulating translation efficiency. These modifications, which are frequently concentrated at the wobble location of tRNAs, have a major influence on the precision of anticodon‐codon interactions, fine‐tuning translation accuracy, and efficiency. RNA modifications exert direct control over codon recognition via particular base and sugar position changes, ensuring translational fidelity and efficiency. These modifications help control the reliability and alacrity of the translation process.^217^, ^218^, ^219^ Modifications in tRNAs can induce substantial structural rearrangements and precise adjustments within the three‐dimensional configuration of tRNAs. This structural flexibility is of paramount importance for tRNAs, enabling their interactions with a diverse array of proteins and other RNA molecules.^220^ It has been observed that the defects in the tRNA may affect the translation process and result in abnormal and defective protein molecules eventually leading to many neurodegenerative disorders.^219^ Moreover, due to genetic mutation, various lethal modifications arise in RNA that further affect the protein expression. These modifications sometimes become a cause of several neurodegenerative disorders along with other diseases. To investigate the role of m6A regulators in PD, a study was conducted on mice that were given dosages of MPTP. There was an obvious upregulation of ALKBH5 and IGF2BP2 along with downregulation of YTHDF1 and FMR1 proteins in the dopamine‐producing region (SN), which shows that m6A regulation could play a role in PD.^221^ RNA editing activities inside exonic regions have a major impact on RNA molecules' translation,subcellular localization, and stability.^168^, ^222^ Emerging evidence suggests that age‐related RNA editing dysregulation, notably at the glutamine/arginine (Q/R) location within glutamate ionotropic receptor AMPA type subunit 2 (GluA2) mRNA can cause excitotoxicity, which is principally caused by an excess of calcium ions (Ca^2+^) influx through Ca^2+^ permeable α‐amino‐3‐hydroxy‐5‐methyl‐4‐isoxazole propionic acid receptors. This process is now recognized as a key mechanism behind motor neuron degeneration in ALS.^223^, ^224^, ^225^

Neurodegenerative disorders are predominantly influenced by the misfolding and aggregation of proteins, presenting appealing avenues for preventing these ailments. For instance, in AD, emerging research indicates that the m6A modification of the tau protein can influence its proclivity for aggregation and the subsequent toxicity it exhibits.^226^, ^227^ As indicated by the research dysregulation in m6A modification may cause translation defects and eventually misfolding of proteins. This misfolding and aggregation of proteins may cause several conditions leading to the development of neurodegenerative disorders.^228^ For example, in AD, the brain experiences the buildup of Aβ and tau proteins, resulting in the formation of plaques and tangles.^229^, ^230^ Likewise, PD involves the accumulation of alpha‐synuclein protein, leading to the development of Lewy bodies in the brain.^231^, ^232^ HD is characterized by the aggregation of mutant huntingtin protein, resulting in intracellular aggregate formation.^233^ Similarly, in ALS, the disease process is associated with the accumulation of misfolded proteins, such as superoxide dismutase 1 (SOD1) and TDP‐43, contributing to the disease's pathogenesis.^234^, ^235^

The age‐related disruption in RNA editing (A‐to‐I) may potentially contribute to the age‐related buildup of circular RNAs (circRNAs) in the brain. These circRNAs could potentially assume a pathogenic role in neurodegenerative conditions like ALS.^225^ circRNAs have also been associated with the regulation of splicing and formation of Muscleblind (MBL) mRNA,^236^ and a study reported that circRNAs were missing from the substantial nigra and at least 24 circRNAs were seen accumulated in the different regions of the brain in PD individuals.^237^ Experiments on mice suffering from Huntington's chorea revealed that there is hypermethylation of m6A along with synapse‐associated genes, it has been shown that m6A was synchronized in a way that was experienced regulated, hence overall it was contributed that differential RNA methylation is related with the learning and cognitive symptoms of the disease.^238^

### 10.3 RNA stability and decay

RNA stability is the ability of RNA molecules to withstand degradation processes. This characteristic of RNA molecules holds paramount importance because it determines the lifespan of RNA molecules, subsequently influencing gene expression and the synthesis of proteins.^239^ RNA stability is a critical factor in many cellular processes. Recent studies have suggested that it maintains the balance between RNA synthesis and decay, which is important in the precise regulation of gene expression.^72^, ^74^, ^240^, ^241^ The stability of mRNA is significantly influenced by the sequence of nucleotides, which impact both the secondary and tertiary structures of the mRNAs and the accessibility of several binding proteins to interact with them.^242^ Additionally, RNA stability is influenced by some other factors as well, including RNA‐binding proteins, RNA alterations, and histone modifications.^240^ For instance, the m1A RNA modification enhances the stability of tRNAs, facilitating translation initiation. Conversely, m1A‐modified mRNAs disrupt Watson‐Crick base pairing with tRNAs, leading to the inhibition of translation.^150^ Furthermore, RNA decay‐related elements like ADAR2 can influence RNA stability by altering the accessibility of RNA‐binding proteins that promote RNA degeneration.^243^ A recent study shows that RNA modifications such as m6A and m5C play an important role in the complicated orchestration of mRNA stability.^241^, ^244^ Modern advancements in high throughput sequencing practices have also provided valuable explanations of the crucial role of mRNA modification and regulation of mRNA nucleotide sequences and stability.^242^ The m6A modification has a regulatory role in different molecular processes for instance transcription, mRNA export, pre‐mRNA splicing, mRNA stability, and translation.^245^, ^246^, ^247^, ^248^ Experiments conducted in vitro, have revealed that mRNAs initiated with m6A modifications show greater resistance to decapping processes catalyzed by mRNA decapping enzyme 2 (DCP2) compared to other forms of mRNA initiation, resulting in increased mRNA stability.^249^, ^250^

In the CNS, RNA stability is carefully controlled through various processes like alternative splicing, and mRNA transport. These mechanisms are essential for the normal functioning of neurons and the brain.^239^ Current studies on transcriptome analysis indicate that noncoding RNAs are substantially expressed in mammalian cells, particularly in the CNS, where they show various expression patterns specific to tissue and cell types. Moreover, these noncoding RNAs have been recognized as intricate and dynamic regulators of multiple signaling pathways linked with neurodegenerative processes.^251^ The precise mechanism through which lncRNA influences the initiation and development of neurodegenerative disorders is not clear until now. In one aspect some research that has been done on lncRNA provided insufficient information on biological replicas or conducted in labs without validation in living organisms. Also, the expression of lncRNA is exceptionally different in patients and normal people, due to a lack of tissue specificity poses a significant challenge. For instance, maternally expressed 3 (MEG3) exhibits show differential expression in HD, AD, and ALS and a few malignant tumors. This makes it challenging to find lncRNAs specific to the CNS for use as potential biomarkers.^252^ Thus, dysregulation of RNA modification can lead to the compromised RNA stability, which eventually disrupts various cellular functions and contributes to the pathogenesis of neurodegenerative disorders.

## 11 EPITRANSCRIPTOME AND IMPORTANT NEURODEGENERATIVE DISEASE

### 11.1 Epitranscriptome and PD

**Figure 4:**
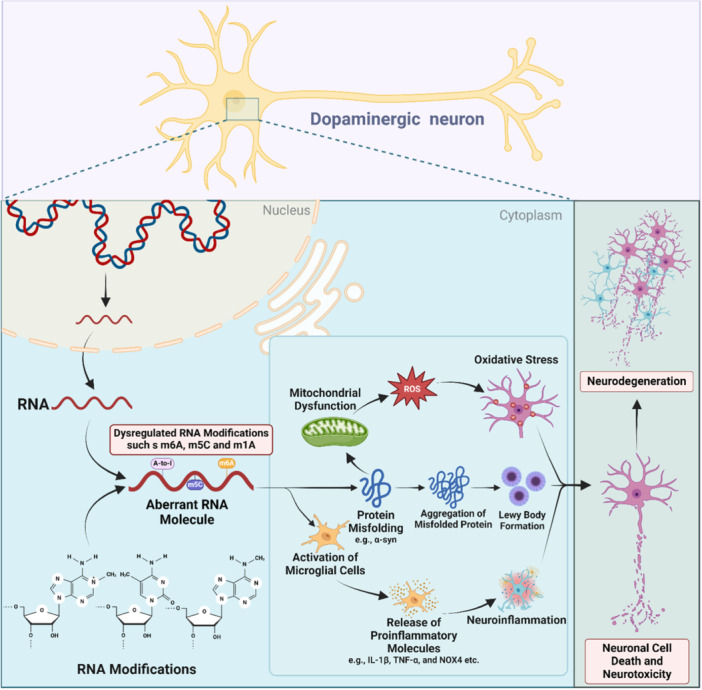
Pathophysiology of PD. PD has been affected by the compromised expression of the various RNA modifications especially m6A, m5C, and A‐to‐I editing. The compromised expression of these specific modifications may induce neurodegeneration by activation of the microglial cells leading to neuroinflammation, protein misfolding that may lead to oxidative stress and Lewy bodies formation, eventually leading to the compromised health of dopaminergic neurons and ultimately leading to the death of these neurons. [Color figure can be viewed at wileyonlinelibrary.com]

PD is a widespread neurodegenerative condition marked by damage to the Brodmann area 9 (BA9) of the brain and the death of dopaminergic neurons, which eventually affects dopamine synthesis.^253^, ^254^, ^255^ PD is characterized by two prominent pathological hallmarks: extensive aggregation of alpha‐synuclein and progressive degeneration of the nigrostriatal system.^256^, ^257^ The disease primarily unfolds due to the aggregation of alpha‐synuclein protein, which culminates in the formation of Lewy bodies within afflicted neurons. The precise origins of alpha‐synuclein aggregation remain enigmatic though.^256^ Various pathological phenomena such as genetic predisposition and environmental factors are associated with PD, but more recently, epitranscriptomic and transcriptomics have also been observed to play an important role in the pathophysiology of PD (Figure 4).^253^, ^258^ There is an indication that m6A alterations are also engaged in the demise of dopaminergic neurons, a characteristic feature of PD.^254^, ^259^ The striatum of PD rat brain and 6‐hydroxydopamine (6‐OHDA)‐induced PC12 cells were used in an investigation. It was found that both the striatum of PD rat brains and 6‐OHDA‐induced PC12 cells exhibited lower levels of global m6A modification of mRNAs. Next, the nucleic acid demethylase FTO was overexpressed or m6A inhibitors were used to lower the levels of m6A in dopaminergic cells. Results indicated that m6A decrease raised the expression of N‐Methyl‐d‐Aspartate (NMDA) receptor 1, raised Ca^2+^ influx, and oxidative stress, all of which ultimately contributed to the dopaminergic neurons' death. This discovery provides new insight into the epigenetic control of PD and reveals that m6A modification is crucial in the death of dopaminergic neurons.^254^ Similarly, studies conducted on the SN and striatum of rat models with PD produced by MPTP showed decreased levels of global m6A modification, further supporting the idea of the involvement of m6A modification in neurodegenerative disorders.^221^

In another study, the expression of m5C and 5‐hydroxymethylcytosine (5‐hmC) in brain tissue samples from PD patients and healthy controls was assessed using the immunohistochemistry technique. The findings revealed substantial increases in 5‐mC expression in the cortical brain regions of PD patients, whereas no variations in the expression of 5‐hmC were observed. Whereas, brain stem and SN exhibited negligible differences in the expression of m5C or 5‐hmC between PD patients and controls. It should be noted that 5‐hmC levels were much higher in the cerebral white matter of PD patients, but m5C levels were not observed to differ. These results suggest that these RNA modifications are thought to have important roles in the etiology of PD.^129^, ^260^ Another RNA modification related to PD is A‐to‐I editing, which is a post‐ or co‐transcriptional change of mRNA nucleotides that may impact RNA structure and function.^253^ In the study, healthy controls and PD patients were analyzed differently for A‐to‐I modifications, and it was discovered that A‐to‐I modifications were significantly reduced in PD patients, as well as altered and abnormal A‐to‐I modifications were also observed, emphasizing the possible role of A‐to‐I modifications in the pathogenesis of PD.^253^

### 11.2 Epitranscriptome and ALS

ALS is a devastating neurodegenerative disease characterized by the progressive degeneration of nerve cells in the brain and spinal cord, primarily affecting upper and lower motor neurons. This leads to muscle weakness, paralysis, and ultimately mortality.^261^ RNA modifications, including structural alterations in RNA‐binding proteins like Fused in Sarcoma (FUS), m6A modification, and A‐to‐I RNA editing, have been linked to the pathophysiology of ALS. These changes can affect RNA stability, transport, and translation, potentially leading to the accumulation of RNA‐binding proteins like TDP‐43, which is a hallmark of ALS pathogenesis.^262^ ADAR2 plays a vital role in a specific RNA editing process, facilitating the conversion of adenosine (A) to inosine (I) at the glutamine/arginine (Q/R) site within GluA2.^263^ This alteration, transforming glutamine into arginine, is indispensable for the proper functioning of AMPA receptors in the adult brain. In mice subjected to conditional ADAR2 knockout, the Q/R site in GluA2 remains unedited, leading to a gradual demise of motor neurons and the onset of motor neuron disease.^104^, ^264^ Recent findings have highlighted a notable reduction in the efficiency of RNA editing at the GluA2 Q/R site in ALS cases compared to healthy controls. Remarkably, among the ADAR family members, only ADAR2 shows a decline in its enzymatic activity within ALS‐afflicted motor neurons. This hints at a critical threshold below which ADAR2 expression levels must not drop, as insufficient editing of GluA2 Q/R sites sets off a sequence of motor neuron degeneration. This ongoing reduction in ADAR2 activity is closely associated with the development of ALS, with the inability to transition A‐to‐I at the GluA2 Q/R site being a key factor.^104^ Additionally, it's noteworthy that ADAR2 primarily governs the editing of the K/E site within CYFIP2 mRNA, representing a novel discovery of ADAR‐mediated A‐to‐I editing. This finding holds the potential to offer valuable insights into the underlying mechanisms driving ALS pathogenesis.^265^

The spinal cords of individuals with ALS exhibit dysregulated m6A modification. Remarkably, m6A modifications are involved in regulating RNA binding by TDP‐43, a protein that undergoes mislocalization from the nucleus in ALS cases. TDP‐43 possesses the capability to recognize m6A‐modified RNA, and RNA methylation is indispensable for both TDP‐43 binding and autoregulation. Moreover, a single‐cell CRISPR/Cas9 candidate‐based screen conducted in primary neurons has identified various m6A‐related factors capable of either enhancing or suppressing TDP43‐mediated toxicity. Among these factors, YTHDF2, a canonical m6A reader, accumulates within spinal motor neurons in postmortem ALS tissue sections. Silencing YTHDF2 has been shown to extend the survival of human neurons carrying ALS‐associated mutations. These findings collectively underscore the pivotal role of m6A modifications in influencing RNA binding by TDP‐43 and in contributing to TDP‐43‐related neurodegeneration in ALS.^266^

### 11.3 Epitranscriptome and AD

AD is a gradual neurodegenerative condition impacting the brain, resulting in memory impairment, cognitive deterioration, and alterations in behavior. The precise etiology of AD remains elusive, but it is identified by the occurrence of irregular protein aggregations in the brain, notably involving Aβ and tau proteins.^267^ These proteins aggregate to create plaques and tangles, disrupting the normal functioning of brain cells. This interference results in the demise of these cells and a gradual loss of brain tissue over time.^268^ Overall, AD is intricate and varied, with its manifestation influenced by a range of factors. Vascular disorders and heightened neuronal excitability are among additional elements that may contribute to the presentation of the disease.^269^ Apart from this, recent investigations propose that epitranscriptomic modifications, specifically m6A and m5C may be involved in the development of AD.^270^, ^271^ O‐linked‐d‐N‐acetylglucosamine (O‐GlcNAc) is a prevalent and distinct posttranslational modification capable of modifying serine and threonine residues in nuclear and cytoplasmic proteins. O‐GlcNAc dysregulation has been associated with a variety of metabolic disorders, including cancer, neurodegeneration, and diabetes.^272^ Small noncoding RNAs known as tRNA‐derived RNA fragments (tRFs) are also associated with a variety of human disorders, which include infectious, metabolic, as well as neurodegenerative disorders.^273^, ^274^ In the hippocampus of AD patients, tRFs produced from a subset of tRNAs are dramatically changed. Some tRFs' altered expression was age‐ and disease‐stage dependent. Angiogenin (ANG) is markedly increased in AD, suggesting that it plays a role in triggering tRFs in AD.^273^, ^274^, ^275^

The CNS, cerebrospinal fluid, and peripheral regions have all been successfully shown to exhibit these modifications, highlighting their promise as biomarkers for AD.^276^ NSUN and TRNA aspartic Acid methyltransferase (TRDMT) protein families are prominent methyltransferases of m5C modifications.^277^ Different studies have observed different expressions of these proteins in control and AD patients. One study found the differential expression patterns of NSUN6 and NSUN7 in AD patients and controls. This highlighted the potential role of 5mC/5hmC and m5C effector proteins in contributing to the emergence of neurodegenerative features associated with AD.^278^ The very important role of NSUN in the m5C methylation and recent discoveries around the involvement of m5C in AD pathogenesis have intrigued scientists to study the possible role of the NSUN protein family in the progression of AD. Mutations in the NSUN2 gene have also been linked to neurological abnormalities. In a study, NSUN2 was observed to be under‐expressed in the brains of patients with AD. In the same study, it was observed that NSUN2 deficiency leads to tau hyperphosphorylation in mouse brains and induced pluripotent stem cells (iPSC)‐derived neurons. Neuronal NSUN2 decreases with Aβ exposure. Overexpressing NSUN2 rescues Aβ‐induced tau phosphorylation and cell toxicity, suggesting a potential therapeutic target for AD.^279^ Another study on circRNAs and synaptic impairment in early‐stage AD investigated the role of m6A in synaptic dysfunction. The study identified upregulated circRIMS2, mediated by METTL3‐dependent m6A modification, which led to synaptic impairments through ubiquitin conjugating enzyme E2 K (UBE2K)‐dependent ubiquitination of the GluN2B subunit of NMDA receptors. This research demonstrated that m6A‐modified circRIMS2 contributes to AD‐related synaptic and memory impairments, highlighting potential therapeutic strategies targeting m6A and associated pathways.^280^

## 12 TARGETING THE EPITRANSCRIPTOME FOR THERAPEUTIC DEVELOPMENT

Epitranscriptomics, a rapidly advancing field, explores chemical modifications in RNA and their influence on gene expression and disease development. Targeting the epitranscriptome has become a promising therapeutic avenue for diverse human diseases, notably neurodegenerative disorders.^281^ This approach can effectively regulate the stability, translation, and localization of crucial disease‐associated mRNAs, thereby influencing disease progression. RNA modification‐based therapeutics aim to intervene by targeting the responsible RNA‐modifying enzymes or factors, rectifying aberrant RNA modifications, or modulating the expression of genes integral to RNA metabolism. Specific RNA‐modifying enzymes intricately regulate the epitranscriptome. Dysregulation in this intricate system has been implicated in the pathogenesis of neurodegenerative disorders, prompting the exploration of epitranscriptomic proteins as potential therapeutic targets for neurodegenerative disorders.^282^ This highlights the potential of intervening at the RNA modification level to address the complexities of neurodegenerative disorders.

### 12.1 RNA‐based therapeutics and epitranscriptomic interventions

RNA‐based therapeutics, a novel class of drugs targeting RNA molecules or pathways, hold promise in treating neurodegenerative disorders such as AD.^283^ Crucial for governing RNA stability, splicing, translation, and localization, RNA modifications can lead to neurodegenerative conditions when dysregulated.^16^, ^271^ RNA‐based therapeutic strategies aim to rectify defective RNA modifications or modulate gene expression involved in RNA metabolism.^284^ Examples of these strategies include antisense oligonucleotides, small interfering RNAs, microRNAs, and aptamers.^284^, ^285^, ^286^ These molecules selectively bind to RNA targets, altering their function or recruiting cellular machinery to degrade or silence them.^284^, ^286^, ^287^ RNA aptamers are single‐stranded RNA molecules that can bind to specific targets, such as proteins or nucleic acids, with high affinity and specificity.^288^ RNA aptamers have emerged as promising theragnostic tools against a wide variety of disorders, including cancer, viral infections, and neurodegenerative disorders.^282^, ^288^, ^289^ RNA aptamers can be used to modulate the RNA metabolism or function in neurodegenerative disorders, such as ALS and spinal muscular atrophy (SMA).^290^, ^291^ As therapeutics, aptamers have great potential because of their ability to bind to proteins and selectively limit their activities with negligible side effects. Aptamers have already been successful in treating various kinds of RNA modifications‐based cancers and this can be replicated in neurodegenerative disorders as well.^292^

Various kinds of RNAs can be targeted by using these RNA‐based therapeutics regarding neurodegenerative disorders. Numerous lncRNA transcripts intricately regulate gene expression across various levels and are heavily influenced by the action of RNA modifications.^293^ Some lncRNAs play a role in the pathogenesis of neurodegenerative disorders by influencing the expression of genes associated with RNA modifications, including writers, readers, and erasers of various RNA modifications. For instance, the upregulation of lncRNA nuclear paraspeckle assembly transcript 1 (NEAT1) in AD interacts with the m6A reader YTHDF2, promoting the degradation of m6A‐modified mRNAs.^294^ Similarly, lncRNA HOX transcript antisense RNA (HOTAIR) is downregulated in PD and affects the pseudouridylation of synuclein alpha (SNCA) mRNA, which encodes alpha‐synuclein, a key protein in PD pathogenesis.^294^, ^295^ A diverse range of RNA therapeutics targeting lncRNAs holds promise for treating neurodegenerative disorders. In recent studies involving AD mouse models, antisense oligonucleotides (ASOs) effectively reduced the expression of lncRNA NEAT1, thereby restoring m6A levels and the translation of m6A‐modified mRNAs like BDNF and APP.^296^ Similarly, miRNAs targeting lncRNA metastasis associated lung adenocarcinoma transcript 1 (MALAT1), showcase the potential in reducing its expression and inhibiting the m6A eraser FTO, associated with ALS. Finally, aptamers targeting lncRNA beta‐secretase 1 (BACE1)‐antisense RNA (BACE1‐AS) demonstrated efficacy in reducing its expression and inhibiting the transcription of BACE1, a key enzyme in AD pathogenesis.^296^ miRNAs, small noncoding RNAs of approximately 22 nucleotides, bind to the 3' UTRs oftarget mRNAs, inhibiting their translation or inducing degradation.^297^ miRNAs also regulate the expression of genes involved in RNA modifications, such as METTL3, FTO, and ADAR1. Dysregulation of such miRNAs may also lead to the dysregulation of any of these enzymes, eventually contributing to the pathogenesis of diseases. In a study, the downregulation of miR‐124 in PD targets FTO, resulted in increased m6A levels and reduced translation of m6A‐modified mRNAs.^298^ RNA modifications impacting mRNA can also contribute to the development of neurodegenerative disorders by influencing local mRNA translation in synapses, a crucial process for synaptic plasticity and memory formation. Enzymes like methyltransferases, PUSs, and deaminases modify synaptic mRNAs, and disruptions in these enzymes can disrupt mRNA function.^298^ For instance, mutations in the pseudouridine synthase PUS1 may decrease pseudouridylation of synaptic mRNAs (e.g., Arc and BDNF), impairing their translation and synaptic plasticity in ALS mouse models.^299^ Similarly, dysregulation of the m6A writer METTL3 can impact m6A modification of synaptic mRNAs (e.g., GluA1 and GluA2), altering their translation and synaptic strength in AD mouse models. Targeting these mechanisms holds potential for developing treatments for various neurodegenerative disorders.^298^

While RNA‐based interventions hold immense potential, their translation from the laboratory to clinical applications encounters substantial challenges. Delivering these RNA‐based therapeutics to target cells in the CNS is impeded by the BBB's selective nature. The BBB restricts the entry of most drugs into the CNS, posing a significant obstacle to effective RNA‐based therapeutic delivery. Additionally, concerns revolve around the specificity and toxicity of these therapeutics, potentially inducing off‐target effects, immune activation, toxicity, and inflammation. Thus, further research and development are imperative to refine the design, delivery, and efficacy of RNA‐based therapeutics for neurodegenerative disorders.^283^

### 12.2 Small molecule modulators of RNA modification enzymes

In recent years, many developmental approaches have been done on mRNA, either to bear out the particular modifications and their characteristics or to target those modifications as a therapeutic strategy in treating various physical or mental disorders.^300^ A normal animal's body works on proteins and proteins are interconnected with each other. As the name indicates small molecular inhibitors are those having low molecular weight and they react with proteins in such a way that the biological activity of that targeted protein reduces and a specific pathway stops, or a specific product starts declining due to its stopped production. Small molecule inhibitors target the association between protein and RNA, specifically RNA binding proteins due to which various RNA modifications arise or they might change.^301^ This section will gather various small molecule inhibitors of RNA modifications that could help treat certain neurodegenerative disorders. METTL3 has a main role in depositing the N‐6 methyl group on the m6A adenosine part that occurs on the mRNA.^302^ A study from Cambridge showed the use of two laboratory‐synthesized METTL3 inhibitors to hinder the protein expression of the compound (RNA modifying enzymes) that have a role in acute myeloid leukemia (AML),^302^ and inference from the study is that if a disease like cancer could be treated, there are possibilities that METTL3 could assist as an agent in treating neurodegenerative disorders. There are some METTL3 inhibitors made from natural products, and some are allosteric, the advanced one is of integrase inhibitor elvitegravir which could be used to treat neurological disorders, even though there is no such study that indicates the specific use of this inhibitor.^303^ Other than that various patent inhibitors provided by pharmaceutical industries are available, some natural products like quercetin, scutellarin, and luteolin are available that have been confirmed by in‐vitro experimentation as inhibitors of METTL3, and they could be obtained to play a role in treating neurodegenerative disorder but still, no proper literature has been cited about it.^303^, ^304^ Integrase inhibitor elvitegravir also intermingles with METTL3, and mechanically it promotes its degradation by coping up interaction with the ubiquitin E3 ligase STIP1 homology and U‐Box containing protein 1 STUB1.^303^, ^305^ For the treatment of AD, the study showed that 24 small molecules have the potential to treat as well as provide neuroprotection against the disease, these were either different modulators and enhancers, others were inhibitors.^306^ METTL3 and ubiquitin specific peptidase 7 (USP7) expressions were interrelated in hepatocellular carcinoma cells and conclusive remarks obtained were that maybe METTL3 can regulate the USP7 expression by m6A methylation.^307^ Neuroinflammation initiated by the microglial cells of the immune system is known as microglial neuroinflammation and it plays a role in neurodegeneration. Microglia neuroinflammation inhibition could be treated with USP7 as it maintains the stability of various proteins in different biological complexes.^308^ USP7 has been identified as a target in treating neurodegenerative as it inhibits microglial activation so that neuroinflammation can be minimized. Oxidative stress is regulated by the Keap1/Nrf2 pathway, targeting them along with USP7 could become a strategy to reduce microglial activation although no clear evidence is present.^308^

As glial cells react to conditions of neuropathology and other brain damage, thus treating the neuroinflammation properly would help reduce the agony due to disease. The FTO, an RNA m6A demethylase, has a positive as well as a negative impact on the nervous system and neurons.^250^, ^309^ FTO RNA demethylases have been shown to induce reversibility of m6A modification.^250^ Two neuroprotective type inhibitors of FTO were synthesized through the computational method and were used and analyzed through an artificial BBB, concluding that it could become a therapeutic strategy in curing AD, PD, etc.^259^ Although FTO's role in neuroinflammation or neurodegeneration has not been confirmed yet, it has a part in the development and functionality of the brain,^196^ and it has been associated with various kinds of neurological disorders like PD, AD, epilepsy, and even depression, and anxiety.^196^ Ten‐Eleven Translocases (TET) is a group of enzymes associated with the conversion of m5C to 5‐hmC. TET proteins could be used in AD and Major depressive disorder (MDD) in the regulation of neurogenesis. TET inhibitors could be used in the control of diseases like Parkinson's, Huntington's, Rett Syndrome, and MDD.^310^ Neuronal death could be a cause of pathological neurological disease, and it is as the disease prevails. It can be prevented through RNA editing, as an experiment showed that reduced RNA editing at the site of GRIA 2 R/G transcripts provided a protective method for the neuronal cells due to overstimulation of Glutamate.^311^ In conclusion, there are many small molecule modulators either in the form of demethylases or translocases, but their efficient use is not enough studied or gaps remain to be investigated. Inhibitors of several enzymes are present but their role in specific RNA modification is yet to be studied.

## 13 CHALLENGES

Scientists have made remarkable progress in the field of epitranscriptomics in recent times by using advanced technologies. Many new approaches are being discovered and existing ones are being improved to enhance the performance. Moreover, many detection methods have better findings but still, there is a problem, it cannot detect multiple modifications in a biological sample or specific RNA molecule at a time.^79^ Epitranscriptomic modification constitutes an additional regulatory layer that governs the gene expression during both physiological and pathological processes of the brain. Despite their limited presence, these modifications undergo active regulation by enzymatic writers and erasers, playing a key role in driving activity‐dependent gene expression within the brain. Moreover, above discussed modifications of the function of various less prevalent alterations including m7G, ac4C, m6Am, and 2′‐O‐methylation (Nm) are currently unfolding, broadening our understanding of the intricate landscape of epitranscriptomic regulation.^300^ A main challenge to explore these modifications is the presence of limited antibodies and chemical agents. Different controversial observations have been reported due to antibody cross‐reactivity issues in the study of epitranscriptomic modification. For instance, this challenge is evident in the cross‐reactivity between the m1A antibody and m7G, introducing a potential source of preference that has influenced previously documented transcriptome‐wide prevalence of m1A.^312^ Similarly, m6A interacts with m6Am adding another layer of complexity to the accurate detection of epitranscriptomic modification.^313^

Furthermore, most of the recent studies are confined to just profiling this modification specifically in CNS diseased models. Generally, overexpression of mouse models and small molecule modules targeting epitranscriptomic enzymes promises to enhance our comprehension of RNA modifications in the context of neurological disorders.^314^ A few years has been focused on viral and nonviral nanocarriers to enhance drug delivery, improving drug solubility, protection from degradation, and circulation times to develop therapeutic efficacy.^315^ BBB is an active and highly specific connection between blood and brain tissue. It acts as a major barrier in brain therapies due to its structure, made up of endothelial cells that are strongly linked together through a tight junction protein, in which the endothelium also controls the astrocytes, neurons, and pericytes.^316^, ^317^ However, a challenge has been overlooked in the design of nanoparticles (NPs) about diffusion in the brain. Once NPs diffuse in the brains, they reach their target through constricted and negatively charged extracellular spaces while evading clearance mechanisms.^318^ Even though the prevalence of CNS disorder increases day by day, CNS drug development remains challenging due to high costs, long pathways to clinical practice, and high failure rates. CNS are intricately shielded by physiological barriers specifically BBB and cerebrospinal fluid barrier, which impose restrictions on the accessibility of the majority of drugs. Biomaterials can be designed to facilitate the control of drug delivery in CNS.^319^ During the previous few years, various strategies have been developed to incapacitate the BBB, by using NPs based on the biomaterials and use of other different approaches to open the BBB.^320^

At present, there are four approved drugs for treating AD. These contain four inhibitors that target acetylcholinesterase and a drug called memantine, which acts as an antagonist for NMDA. Aside from these medications, which have limited clinical trials, the 4‐decade‐long quest for effective treatment of AD has faced a 100% failure rate in clinical trials. These include compounds that aim to address brain amyloid deposition or eliminate amyloid plaques and other putative disease‐linked processes.^321^ Involvement of Rho Kinase (ROCK) signaling pathways provides a promising for the development of new therapeutic strategies.^322^ In the early stage of research, there are now diverse and relevant models available. This helps researchers to test and confirm proposed treatment by using different models before clinical trials, reducing the risk of failure due to lack of ineffectiveness.^261^ There is strong evidence suggesting that nuclear receptors (NRs) could be helpful to cure these types of disorders. NR ligand switches on the transcriptional activators that control the expression of a variety of genes that are associated with inflammation and metabolism. While using these activations of NRs in animal models to treat neurodegenerative disorder has shown promising results, in clinical practice, this strategy has been not successful.^323^

## 14 CONCLUSION

In conclusion, the paramount importance of RNA modifications, particularly in the realm of neurodegenerative disorders, has surfaced prominently, emphasizing their substantial influence on gene expression and introducing potential avenues for therapeutic applications. The intricate relationship between RNA modifications and neurodegenerative disorders has become increasingly evident, necessitating further exploration to decipher their nuanced impact on disease progression. Recognizing the pivotal role of this complex network in the pathogenesis of neurodegenerative disorders prompts a deeper dive into the molecular mechanisms governing these intricate interactions.

The promising therapeutic potential inherent in RNA‐based interventions and small molecule modulators targeting specific RNA modification enzymes, such as METTL3 and FTO, has emerged as a beacon of hope in the pursuit of effective treatments. However, persistent concerns about the specificity, toxicity, and potential off‐target effects of these interventions underscore the imperative need for further research and refinement in design and delivery methodologies. As we navigate through the intricate landscape of RNA modification, the ongoing quest for effective therapeutic approaches in neurodegenerative disorders continues.

## AUTHOR CONTRIBUTIONS

Muhammad Abu Talha Safdar Hashmi contributed to the study's conception and design; Hooriya Fatima, Sadia Ahmad, Amna Rehman, Fiza Safdar, and Muhammad Abu Talha Safdar Hashmi were responsible for literature search, data collection, and visualization; Muhammad Abu Talha Safdar Hashmi, Hooriya Fatima, Sadia Ahmad, Amna Rehman, and Fiza Safdar completed the first draft of the manuscript; Muhammad Abu Talha Safdar Hashmi, Hooriya Fatima, and Sadia Ahmad revised the final version of the manuscript. All authors read and approved the final manuscript.

## CONFLICT OF INTEREST STATEMENT

The authors declare no competing interests associated with the publication of this review article.

## ETHICS STATEMENT

Not applicable.

## Data Availability

Not applicable as the manuscript does not contain novel data, and all the information provided can be sourced from literature.
